# Synthesis and UV-Curing Modification of the High cis-1,4-Hydroxyl-Terminated Polybutadiene Binder Suitable for Ultra-Low Temperature Applications

**DOI:** 10.3390/polym18091095

**Published:** 2026-04-30

**Authors:** Hu Lyu, Lei Wang, Yue Li, Guoliang Yu, Shudi Liu, Dongzhou Sun, Yuling Liang, Pengfei Huo, Dawei Zhang, Zhiqiang Ning, Xianzhi Kong

**Affiliations:** 1 Institute of Petrochemistry, Heilongjiang Academy of Sciences, Harbin 150040, China; haitun@hipc.org.cn (H.L.); yuguoliang@hipc.org.cn (G.Y.); liushudi@hipc.org.cn (S.L.); sundongzhou@hipc.org.cn (D.S.); 2 AVIC Shenyang Aircraft Design and Research Institute, Shenyang 110035, China; 13998278321@139.com; 3 Engineering Research Center of Advanced Wooden Materials (Ministry of Education), Northeast Forestry University, Harbin 150040, China; liangyuling@nefu.edu.cn (Y.L.); huopengfei@nefu.edu.cn (P.H.)

**Keywords:** cis-1,4-polybutadiene, ultra-low temperature, oxidative cleavage, propellant binder, 3D printing

## Abstract

As space exploration activities and strategic deployments in polar regions continue to advance, higher demands have been placed on the low-temperature resistance of propellant binders. Here, high cis-1,4 content hydroxyl-terminated polybutadiene (HTPB) was successfully synthesized via an oxidative cleavage method using commercial cis-polybutadiene (BR). The microstructure, molecular weight, hydroxyl value, rheological behavior, thermal properties, and mechanical performance of the resulting cis-HTPB were systematically characterized. By adjusting the molar ratio of mCPBA to butadiene units, three cis-HTPB samples with varying molecular weights were obtained. The high cis-1,4 structure (93%) was preserved after modification. The synthesized cis-HTPB exhibited an ultra-low glass transition temperature (Tg) of approximately −100 °C and lower viscosity compared to commercial HTPB, indicating excellent low-temperature flexibility and processability. In addition, the cis-HTPB was further modified with acrylate groups to produce a UV-curable derivative (AcTPB). The cured AcTPB also retained a Tg near −100 °C, demonstrating its suitability for ultra-low-temperature applications and its promise as a photocurable binder for 3D printing propellant.

## 1. Introduction

Hydroxyl-terminated polybutadiene (HTPB) binders are the most widely used binders in composite solid propellants due to their low cost and high chemical stability [1,2]. Currently, most HTPBs are prepared via free-radical polymerization, with a glass transition temperature (Tg) of approximately −80 °C, which meets general application requirements [3,4]. However, with the expansion of strategic deployments in polar regions and extensive space exploration activities, higher demands have been placed on the low-temperature resistance of solid propellant binders [5]. A lower Tg enables the propellant to maintain flexibility even under extremely low temperatures, thereby reducing the risk of debonding and cracking.

The Tg of HTPB is closely related to the content of cis-1,4, trans-1,4, and 1,2-vinyl units within the polymer [6,7,8]. A higher proportion of cis-1,4 units results in a lower Tg [9]. Currently, the cis-1,4 content of HTPB prepared by free-radical polymerization and anionic polymerization is generally around 40% [3]. Although coordination polymerization can produce polybutadiene rubber with a cis-1,4 content exceeding 95%, existing catalytic systems cannot directly yield HTPB, as nickel compounds or alkylating agents lack hydroxyl groups in their structures at a technical level [4,5]. Zhou et al. addressed this by preparing a series of HTPBs with high cis-1,4 content through the oxidative cleavage of commercial cis-polybutadiene rubber (BR) [10,11,12]. This method is not only simple and cost-effective but also yields HTPB rubber with a cis-1,4 content of approximately 95% and a Tg near −105 °C.

With the advancement of 3D printing technology, an increasing number of countries are utilizing 3D printing for the preparation of solid propellants [13,14,15]. However, to the best of our knowledge, current research on high cis-polybutadiene has primarily focused on synthesis methods and material properties, with no reports available on its photocuring modification or application in 3D printing. Therefore, it is necessary to investigate the performance differences in high cis-polybutadiene before and after UV-curing modification, as well as its potential for 3D printing applications.

In this study, we prepared a series of HTPB samples with high cis-1,4 content (cis-HTPB) and varying molecular weights via the oxidative cleavage method. The properties of the synthesized cis-HTPB were compared with those of commercial HTPB. More notably, we further modified the cis-HTPB by acryloyl chloride termination to obtain UV-curable cis-polybutadiene (AcTPB). Subsequently, the photocuring performance of AcTPB and its potential application in 3D printing were investigated. This work provides a preliminary exploration of the preparation process and properties of UV-curable modified high cis-polybutadiene, offering foundational data for its potential application in 3D printing.

## 2. Materials and Methods

### 2.1. Materials

High cis-1,4-polybutadiene (rubber block, BR) was purchased from Sinopec Yanshan Petrochemical Company (Beijing, China). 3-Chloroperoxybenzoic acid (85%, mCPBA), periodic acid (Ar, H_5_IO_6_), tetrahydrofuran (AR, THF), and cyclohexane (AR) were obtained from Shanghai Macklin Biochemical Co., Ltd. (Shanghai, China). Sodium bicarbonate (AR, NaHCO_3_) was supplied by Tianjin Kermel Chemical Reagent Co., Ltd. (Tianjin, China). Sodium borohydride (AR, NaBH_4_) and triethylamine (AR) were acquired from Tianjin Fuyu Fine Chemical Co., Ltd. (Tianjin, China). Anhydrous ethanol (AR) was purchased from Tianjin Tianli Chemical Reagent Co., Ltd. (Tianjin, China). Acryloyl chloride (96%) was obtained from Shanghai Aladdin Biochemical Technology Co., Ltd. (Shanghai, China). Methyl methacrylate (MMA, 99%), Isobornyl acrylate (IBOA, 98%) and 4-Acryloylmorpholine (ACMO, 98%) were obtained from Shanghai Macklin Biochemical Co., Ltd. (Shanghai, China). All chemicals were used as received without further purification unless otherwise stated.

### 2.2. Synthesis of High cis-Hydroxyl-Terminated Polybutadiene (cis-HTPB)

The synthesis of high cis-hydroxyl-terminated polybutadiene (cis-HTPB) primarily involves three steps: the synthesis of epoxidized polybutadiene (EBR), followed by the synthesis of high cis-aldehyde-terminated polybutadiene (ATPB), and finally the synthesis of high cis-hydroxyl-terminated polybutadiene (cis-HTPB). The entire reaction process was conducted in a 1000 mL three-necked flask equipped with a N_2_ protection system, an oil bath, and a mechanical stirrer.

I. The synthesis of EBR (epoxidation): BR (20 g) was dissolved in 400 mL of a mixed solvent of THF and cyclohexane (1:1 *v*/*v*). The mixture was heated to 30 °C. Subsequently, mCPBA dissolved in THF was added dropwise to the three-necked flask (3 s per drop) using a dropping funnel. After the addition was complete, the epoxidation reaction was continued for 6 h.

II. The synthesis of ATPB (oxidative cleavage): H_5_IO_6_ was dissolved in THF and added dropwise in situ to the EBR solution prepared in the first step (3 s per drop). After the addition was complete, the reaction was continued for 6 h. Subsequently, NaHCO_3_ was added, and the mixture was allowed to stand overnight. The following day, the reaction mixture containing the ATPB was filtered under suction to remove the precipitate at the bottom of the liquid. The crude ATPB product was then obtained by rotary evaporation. After repeated washing with ethanol, the product was dried in a vacuum oven at 30 °C to constant weight, yielding a colorless transparent or light-yellow ATPB sample.

**Figure 1:**
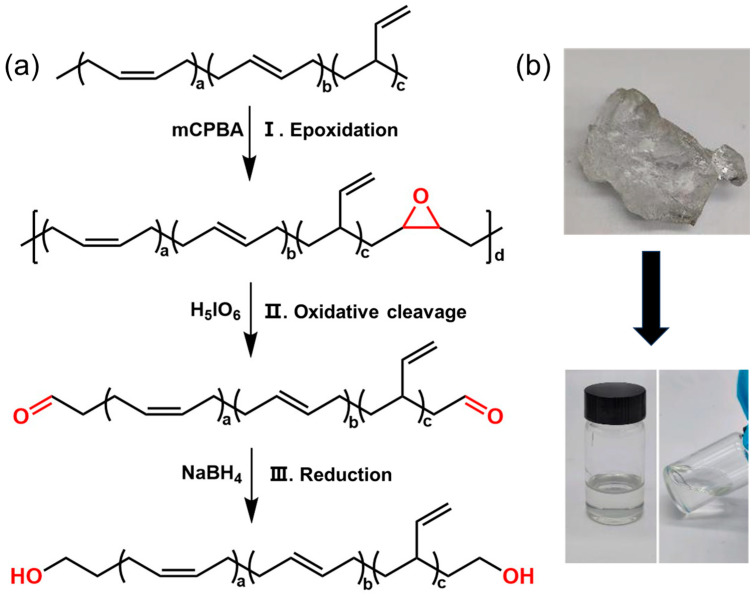
The synthetic route and photo of Cis-HTPB. (**a**) synthesis route of cis-HTPB, (**b**) the photo of the raw material BR and cis-HTPB.

III. The synthesis of cis-HTPB (reduction): ATPB (10 g) was dissolved in 200 mL of a mixed solvent of THF and cyclohexane (1:1 *v*/*v*). After heating to 30 °C, NaBH_4_ and H_2_O were added sequentially (with a molar ratio of NaBH_4_:H_2_O:–CHO in ATPB of 2:2:1). The reaction was allowed to proceed for 8 h, after which a small amount of H_2_O was added again to quench the sodium borohydride. The mixture was then allowed to stand overnight. On the next day, the solvent was removed by rotary evaporation, and the product was redissolved in cyclohexane, followed by centrifugation to remove impurities. Finally, cyclohexane was removed by rotary evaporation, and the product was washed with ethanol and dried in a vacuum oven at 30 °C to constant weight, yielding a colorless transparent or light-yellow cis-HTPB. Figure 1a shows the synthesis route of cis-HTPB. Figure 1b is the photo of the raw material BR and cis-HTPB. 

Three cis-HTPB samples with different molecular weights were prepared by adjusting the molar ratio of mCPBA to butadiene units, and their specific contents are shown in Table 1.

### 2.3. Synthesis of UV-Curable High cis-Hydroxyl-Terminated Polybutadiene (AcTPB)

Cis-HTPB (5 g) was dissolved in THF (100 mL), with triethylamine added as an acid scavenger. The mixture was placed in an ice-water bath. Subsequently, acryloyl chloride (the molar ratio of –OH to –COCl was 1:1) was dissolved in THF and added dropwise at a rate of 3 drops per second. After the dropwise addition, the mixture was heated to 40 °C and reacted for 6 h. Upon completion of the reaction, triethylamine salts were removed by suction filtration, and the solvent was evaporated under reduced pressure. The product was then dried in a vacuum oven at 30 °C to a constant weight to obtain UV-curable AcTPB. After mixing with the photoinitiator, AcTPB can be cured under UV light.

### 2.4. 3D Printing

First, the AcTPB resin was weighed, and the diluent (MMA, IBOA or ACMO) and photoinitiator 819 (2 wt.%) were subsequently added in a predefined proportion. The mixture was magnetically stirred for 30 min to guarantee homogeneous mixing of all components. Finally, high vacuum was applied to remove air bubbles within the ink, and the resulting ink was reserved for 3D printing.

3D printing was implemented using a custom-built extrusion-based 3D printer. The device consists of a precision positioning system, an ink extrusion system, and integrated hardware and software interfaces. The nozzle diameter and extrusion pressure of the printer can be adjusted. The laser (LR-MFJ-405; Changchun Laser Technology Co., Ltd., Changchun, China) operates at a wavelength of 405 nm and delivers an output power of 300 mW.

### 2.5. Characterization Methods

#### 2.5.1. Structure Characterization

Fourier transform infrared spectroscopy (FTIR) measurements were performed using a TENSOR II (Bruker, Berlin, Germany), with 32 scans acquired over the wavenumber range of 400 to 4000 cm^−1^ (resolution: 4 cm^−1^). ^1^H nuclear magnetic resonance (^1^H NMR) and ^13^C nuclear magnetic resonance (^13^C NMR) were conducted on an AVANCE III HD 500 MHz (Bruker, Fällanden, Switzerland) instrument using CDCl_3_ as the solvent.

#### 2.5.2. Gel Permeation Chromatography (GPC)

The molecular weight (Mn) and polydispersity index were obtained by GPC on a PL-GPC 50 (Agilent, Santa Clara, CA, USA) by using THF as eluent solvent at a flow rate of 1 mL/min at 30 °C.

#### 2.5.3. Hydroxyl Value

The hydroxyl value of the cis-HTPB was determined using the acetic anhydride-pyridine method in accordance with the GJB 1372A-2003 [16].

#### 2.5.4. Rheology

The viscosity of cis-HTPB was determined using a rotational rheometer equipped with a parallel-plate rotor. The measurement was performed in frequency sweep mode over the range of 1–100 Hz.

#### 2.5.5. Glass Transition Temperature (Tg)

Tg of cis-HTPB was conducted on a differential scanning calorimeter (DSC214, NETZSCH, Selb, Germany) in the range of −160 to 20 °C at a heating rate of 5 °C min^−1^ under N_2_ atmosphere.

#### 2.5.6. Dynamic Thermomechanical Properties (DMA)

The dynamic thermomechanical properties of samples were measured on a DMA-242E (NETZSCH, Selb, Germany) in tensile mode at 1 Hz with an amplitude of 20 μm. The temperature was increased from −160 to 100 °C at a heating rate of 5 °C/min.

#### 2.5.7. Thermal Stability

Thermogravimetric analysis (TGA) of samples (approximately 10 mg) was characterized by TG209 F3 (NETZSCH, Selb, Germany) under N_2_ atmosphere. The scanning range was 30 °C to 600 °C at a heating rate of 10 °C/min.

## 3. Results and Discussion

### 3.1. Chemical Structure of cis-HTPB

**Figure 2:**
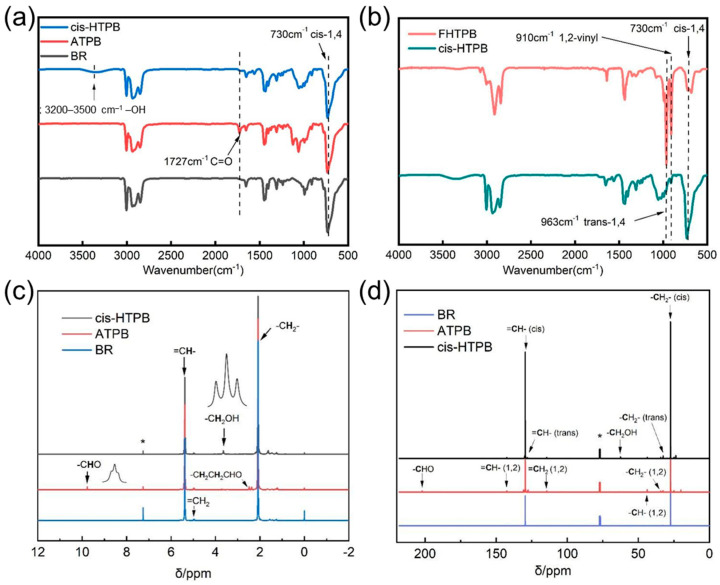
Chemical structure of BR, ATPB, and cis-HTPB. (**a**) FTIR spectra of cis-HTPB, ATPB, and BR; (**b**) FTIR spectra of FHTPB and cis-HTPB; (**c**) ^1^H NMR spectra of BR, ATPB, and cis-HTPB, * is the proton peak of CDCl_3_; (**d**) ^13^C NMR spectra of BR, ATPB, and cis-HTPB, * is the proton peak of CDCl_3_.

Figure 2a shows the FTIR spectra of BR, ATPB, and cis-HTPB. Compared with BR, the spectrum of ATPB exhibits a C=O characteristic peak at 1727 cm^−1^, indicating the presence of –CHO in ATPB. In contrast to ATPB, the spectrum of cis-HTPB shows the disappearance of the C=O peak at 1727 cm^−1^, accompanied by the appearance of a –OH characteristic peak in the 3300–3600 cm^−1^ range [17,18]. This suggests that the aldehyde groups (–CHO) have been reduced to –OH, confirming the successful synthesis of cis-HTPB. Due to their high cis-1,4 content, BR, ATPB, and cis-HTPB all exhibit a strong characteristic peak for the cis-1,4 structure at 730 cm^−1^.

Figure 2b shows a comparison of the FTIR spectra of cis-HTPB and commercial hydroxyl-terminated polybutadiene (FHTPB, cis-1,4:trans-1,4:1,2-vinyl = 20%:60%:20%) prepared by free-radical polymerization. The cis-1,4 unit peak (730 cm^−1^) of cis-HTPB is significantly stronger than that of FHTPB, while the trans-1,4 unit peak (963 cm^−1^) and the 1,2-vinyl unit peak (910 cm^−1^) are notably weaker. These results also demonstrate that cis-HTPB possesses a high cis-1,4 unit content.

Figure 2c shows the ^1^H NMR spectra of BR, ATPB, and cis-HTPB. The proton peak at a chemical shift of δ = 5.38 ppm corresponds to the =CH– protons in the 1,4-units; the proton peak at δ = 4.98 ppm is assigned to the =CH_2_ protons in the 1,2-vinyl units [19]. The proton peak at δ = 2.09 ppm is attributed to the –CH_2_– protons in the main chain. The spectrum of ATPB exhibited a proton peak corresponding to the –CHO at δ = 9.77 ppm, confirming the successful synthesis of ATPB. In the spectrum of cis-HTPB, the proton peak of the –CHO at δ = 9.77 ppm disappears, while a proton peak corresponding to the methylene group (–CH_2_OH) linked to the –OH appears in the range of δ = 3.63–3.67 ppm. This indicates that the –CHO has been reduced to –OH, confirming the successful synthesis of cis-HTPB. In addition, the proton peak of the methylene group (–CH_2_OH) linked to the –OH appears as a triplet, indicating that the –OH groups are primary hydroxyl groups, which means that the oxidative cleavage and chain scission occur at the 1,4-unit of the molecular chain.

Figure 2d shows the ^13^C NMR spectra of BR, ATPB, and cis-HTPB. The characteristic peaks at δ = 27.43 ppm and δ = 129.60 ppm are assigned to the [–CH_2_–(cis)] and [=CH–(cis)] in the cis-1,4 units, respectively; the characteristic peaks at δ = 32.75 ppm and δ = 129.27 ppm are attributed to the [–CH_2_–(trans)] and [=CH–(trans)] in the trans-1,4 units, respectively; the characteristic peaks at δ = 34.31 ppm and δ = 43.79 ppm correspond to the [–CH_2_–(1,2)] linked to the double bond and [–CH–(1,2)] in the 1,2-vinyl units, respectively; and the characteristic peaks at δ = 114.47 ppm and δ = 142.56 ppm are assigned to the [=CH_2_(1,2)] and [=CH–(1,2)] in the 1,2-vinyl units, respectively [19]. The characteristic peak at δ = 202.04 ppm is attributed to the –CHO and appears only in the spectrum of ATPB. Upon reduction of ATPB to cis-HTPB, the peak at δ = 202.04 ppm corresponding to the –CHO disappears, and a peak at δ = 62.53 ppm corresponding to the –CH_2_OH emerges.

The microstructure ratios of the main chains of BR and cis-HTPB were calculated based on the ^1^H NMR spectra and ^13^C NMR spectra. For BR, the ratio of cis-1,4 units to trans-1,4 units to 1,2-vinyl units was 95:3:2. For cis-HTPB, this ratio was 93:5:2. The proportion of the cis-1,4 unit in both polymers showed little change, indicating that the oxidative cleavage process does not significantly alter the microstructure of the rubber main chain. The content of the trans-1,4 units in cis-HTPB increased slightly; the reason may be that the cis-1,4 unit accounted for 95% of BR, and the probability of selecting the cis-1,4 unit as the chain scission site was higher during oxidative cleavage, leading to a slight decrease in the proportion of the cis-1,4 unit in cis-HTPB.

### 3.2. Molecular Weight, Hydroxyl Value, and Viscosity of cis-HTPB

**Figure 3:**
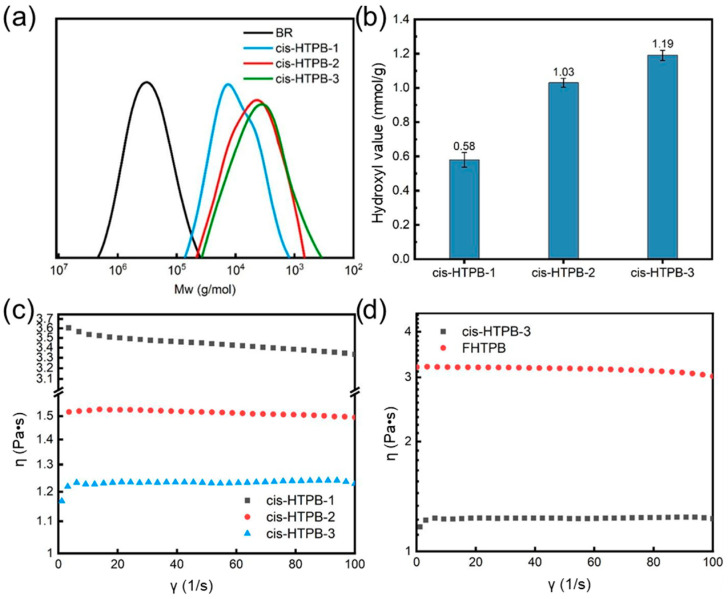
Molecular weight, hydroxyl value, and viscosity of BR and three cis-HTPB samples. (**a**) The GPC curves of BR and three cis-HTPB samples. (**b**) The hydroxyl values of three cis-HTPB samples. (**c**) The viscosity curves of three cis-HTPB samples. (**d**) The viscosity curves of cis-HTPB-3 and FHTPB.

Figure 3a shows the GPC curves of BR, cis-HTPB-1, cis-HTPB-2, and cis-HTPB-3. The mCPBA content can affect the molecular weight of the prepared cis-HTPB. With the increase in the molar ratio of mCPBA to butadiene units, the number of epoxidation and oxidative cleavage sites in the BR main chain gradually increases, resulting in a lower molecular weight of the prepared cis-HTPB. The specific molecular weight information is presented in Table 2. Owing to the inherently high polydispersity index (PDI = 3.51) of the raw BR material, and the random nature of oxidative cleavage reactions, the resulting cis-HTPB consequently exhibits a similarly high PDI (2.84–3.00).

The hydroxyl values of cis-HTPB-1, cis-HTPB-2, and cis-HTPB-3 were 0.58 mmol/g, 1.03 mmol/g, and 1.19 mmol/g, respectively (Figure 3b). As the oxidizing agent content increased, more oxidative cleavage sites were generated on the BR, leading to progressively higher hydroxyl values in the resulting cis-HTPB samples.

The viscosity of a polymer is a critical factor affecting its processability. Figure 3c presents the viscosity curves of the prepared cis-HTPB as a function of shear rate. As the molecular weight of the samples decreased, the viscosity of the samples also gradually decreased. The viscosities of cis-HTPB-1, cis-HTPB-2, and cis-HTPB-3 were approximately 3.5 Pa·s, 1.5 Pa·s, and 1.23 Pa·s, respectively.

Furthermore, a comparison of the viscosities of FHTPB (W_n_: 1700–2200) and cis-HTPB-3 (W_n_: 2187) revealed that the viscosity of cis-HTPB-3 (1.23 Pa·s) was significantly lower than that of FHTPB (3.17 Pa·s), indicating better fluidity (Figure 3d). This difference is likely attributable to the distinct molecular microstructures: FHTPB possesses a higher content of 1,2-vinyl units, which introduce more branched chains, whereas cis-HTPB has a higher proportion of cis-1,4 structures, thereby enhancing the flexibility of the molecular chains.

### 3.3. Tg and Thermal Stability of cis-HTPB

**Figure 4:**
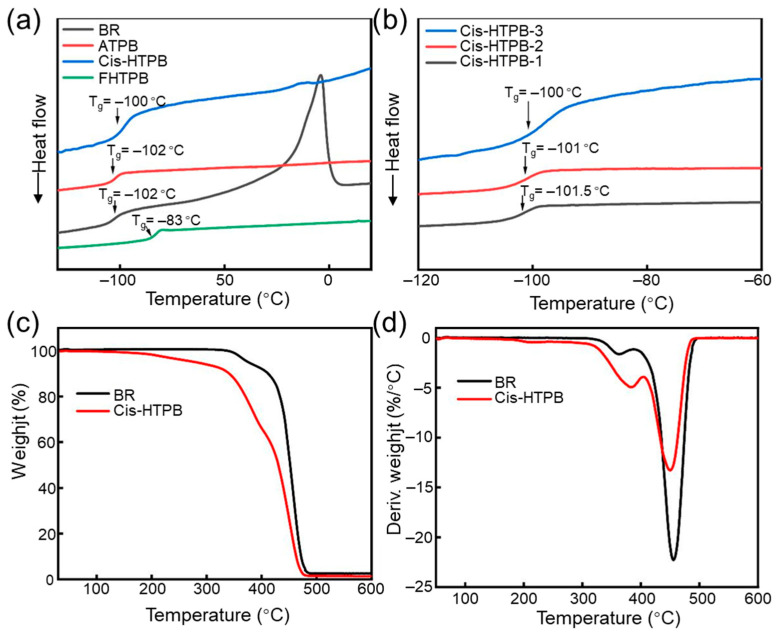
T_g_ and thermal stability. (**a**) The DSC curves of BR, ATPB, cis-HTPB, and FHTPB; (**b**) the DSC curves of three cis-HTPB samples; (**c**) TG curves of BR and cis-HTPB; (**d**) DTG curves of BR and cis-HTPB.

The T_g_ of the polymer directly affects the service temperature range of the material. Figure 4a shows the DSC curves of BR, ATPB, cis-HTPB, and FHTPB. Polybutadiene rubbers (BR, ATPB, cis-HTPB) with a high cis-1,4 unit content exhibit a T_g_ of approximately −100 °C, which is significantly lower than that of FHTPB (−83 °C), resulting in a wider applicable temperature range. Figure 4b shows the DSC curves of three cis-HTPB samples with different molecular weights and hydroxyl values. The T_g_ values of cis-HTPB-1, cis-HTPB-2, and cis-HTPB-3 are −101.5 °C, −101 °C, and −100 °C, respectively, which increase slightly with the decrease in molecular weight and the increase in hydroxyl value. This trend is attributed to the enhanced intermolecular hydrogen bonding interactions resulting from the higher –OH content, which leads to an elevation in the T_g_.

Figure 4d showed the derivative thermogravimetric (DTG) curves of BR and cis-HTPB. BR and cis-HTPB exhibit similar decomposition trends; however, the thermal stability of cis-HTPB is slightly reduced, as seen in the thermogravimetric (TG) curves (Figure 4c). This decrease is attributed to the presence of a small amounts of low-molecular-weight species generated from the oxidative cleavage of BR macromolecules during the synthesis of cis-HTPB.

### 3.4. Mechanics of cis-HTPB

**Figure 5:**
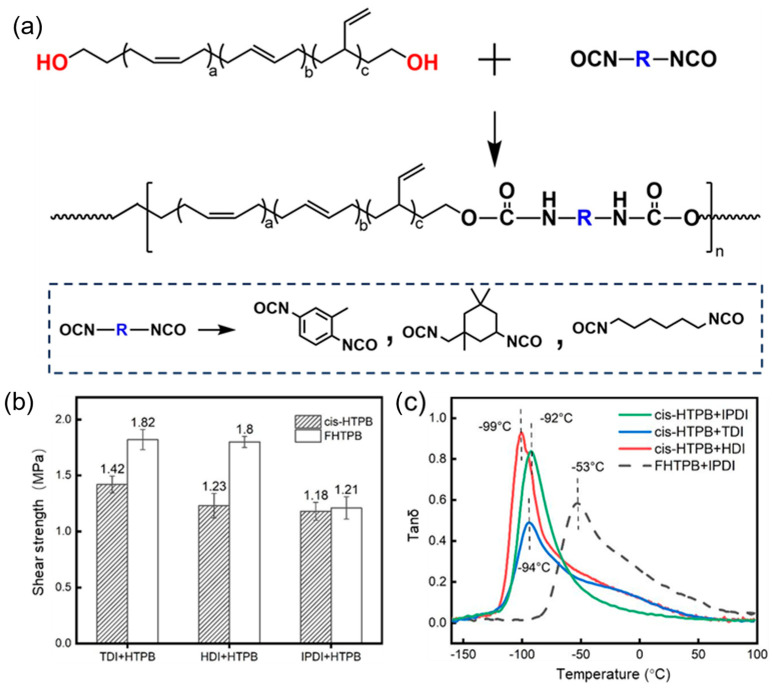
Mechanics of cis-HTPB. (**a**) Schematic diagram of curing mechanism of cis-HTPB. (**b**) Shear strength of cis-HTPB and FHTPB. (**c**) Tanδ curves of cis-HTPB and FHTPB.

HTPB is commonly used as a binder in solid propellants, where it typically undergoes curing by reacting with diisocyanate curing agents. The reaction scheme between HTPB and diisocyanate is shown in Figure 5a. Commonly used isocyanates include HDI (hexamethylene diisocyanate), TDI (toluene diisocyanate), and IPDI (isophorone diisocyanate).

Figure 5b compares the mechanical strength of cis-HTPB-3 and FHTPB, each cured with three different diisocyanates. Although the mechanical strength of the cis-HTPB-3 is slightly lower than that of FHTPB, all values exceed 1 MPa, indicating that cis-HTPB-3 still possesses good mechanical properties.

Furthermore, the Tg of the cis-HTPB-based adhesive was close to −100 °C, significantly lower than that of FHTPB (−53 °C), indicating a broader applicable temperature range (Figure 5c).

### 3.5. Chemical Structure and Properties of AcTPB

**Figure 6:**
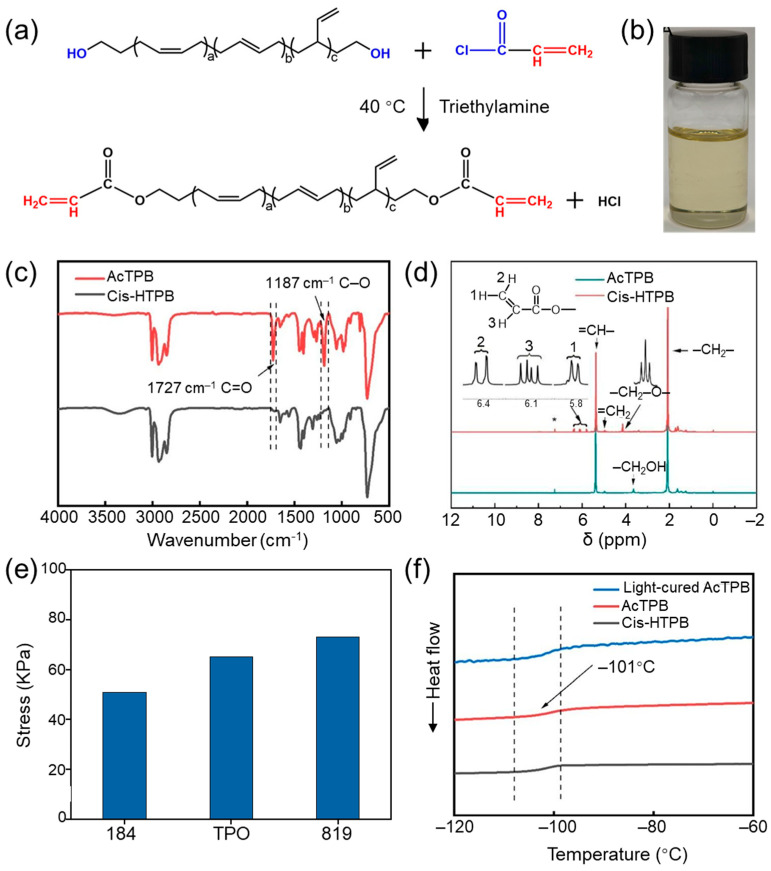
Chemical structure and properties of AcTPB. (**a**) The synthetic route of AcTPB. (**b**) AcTPB samples. (**c**) FTIR spectrum. (**d**) ^1^H NMR spectra. * is the proton peak of CDCl_3_. (**e**) Stress. (**f**) DSC curves of cured AcTPB.

The photocurable high cis-polybutadiene (AcTPB) is prepared by the esterification reaction between the -OH of cis-HTPB-3 and the –COCl of acryloyl chloride, and the reaction route is shown in Figure 6a. AcTPB presents a light yellow transparent state (Figure 6b). After the reaction of cis-HTPB with acryloyl chloride, the -OH peak in the 3300–3600 cm^−1^ region of the AcTPB spectrum essentially disappeared. Meanwhile, the C=O peak appeared at 1727 cm^−1^, and the –C–O–peak emerged at 1187 cm^−1^ (Figure 6c) [20,21]. This indicates that the acrylate groups are successfully grafted onto the cis-HTPB, which initially confirms the successful synthesis of AcTPB.

Figure 6d shows the AcTPB ^1^H NMR spectra. Compared with cis-HTPB, the ^1^H NMR spectrum of AcTPB shows three additional proton peaks at chemical shifts of δ = 5.78–5.82 ppm, δ = 6.08–6.14 ppm, and δ = 6.36–6.41 ppm. These three proton peaks are all attributed to the protons on CH_2_=CH– in AcTPB [22,23]. The proton peak of AcTPB at δ = 4.12–4.16 ppm is attributed to the –COO–CH_2_–, which does not appear in the spectrum of cis-HTPB-3. Meanwhile, the proton signal at δ = 3.63–3.67 ppm in cis-HTPB-3, corresponding to the –CH_2_OH, almost disappears in the AcTPB spectrum. These results demonstrate that -CH=CH_2_ was successfully introduced onto the cis-HTPB-3, confirming the successful synthesis of UV-curable AcTPB.

Upon mixing with various commonly used photoinitiators (819, TPO and 184), the UV-curable resin can be initiated and cured, exhibiting a mechanical strength in the range of 50–75 kPa (Figure 6e). Compared to the cured cis-HTPB, the mechanical strength is slightly lower, which may be attributed to the absence of hard segments (–NH–COO–) in the system.

The Tg of the AcTPB and cured AcTPB remained consistent with that of cis-HTPB-3, at approximately −101 °C (Figure 6f), indicating that AcTPB retains excellent low-temperature performance and remains suitable for use under low-temperature conditions.

### 3.6. 3D Printing Process of AcTPB

**Figure 7:**
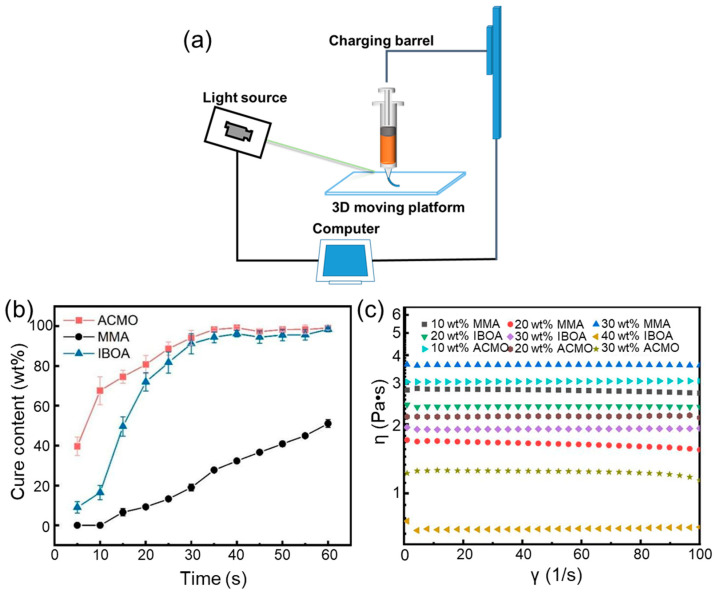
3D printing process of AcTPB. (**a**) The working principle of the extrusion-based 3D printer. (**b**) Curing content of AcTPB mixed with different diluents. (**c**) Viscosity of AcTPB mixed with different diluents.

We also employed an extrusion-based 3D printing process to investigate the printability of AcTPB [24,25,26,27]. Figure 7a illustrates the working principle of the extrusion-based 3D printer. AcTPB exhibits good compatibility and can be mixed with various diluents (MMA, IBOA, ACMO) for UV curing (Figure 7b). The viscosity of the printing ink can be adjusted by varying the diluent content (Figure 7c).

**Figure 8:**
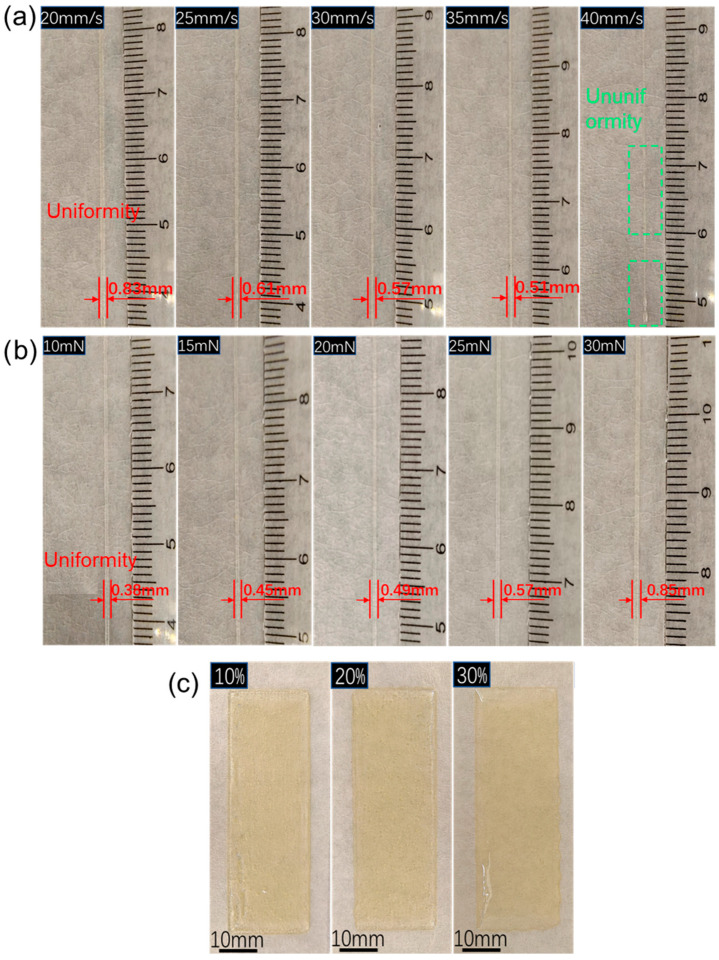
3D printing quality. (**a**) Printing quality of single filament at different printing speeds. (**b**) Printing quality of single filament at different printing pressures. (**c**) Printing quality of rectangular samples with three layers of thickness printed under different diluent contents.

The printing parameters were optimized by examining the printed filaments. The effect of the printing speed of the needle on the printing quality was also studied (Figure 8a). When the printing speed of the needle was too fast (≥40 mm/s), the printed monofilaments were discontinuous with extremely poor quality; when the moving speed was too slow, the diameter of the printed monofilaments was significantly larger than the needle diameter (0.5 mm). When the printing speed was 35 mm/s, the diameter of the printed monofilaments was closest to the needle diameter, indicating relatively good printing accuracy.

In addition, the effect of printer extrusion pressure on the printing quality was investigated (Figure 8b). The results showed that when the extrusion pressure was in the range of 10–30 mN, continuous and uniform monofilaments could be printed. However, the diameter of the printed monofilaments increased with the increase in extrusion pressure. Among them, when the extrusion pressure was between 20 and 25 mN, the diameter of the printed monofilaments was closest to the needle diameter (0.5 mm), indicating the best printing accuracy.

Finally, we printed three layers of rectangular samples with different diluent contents under the optimal parameters (20 mn; 35 mm/S), and the printed samples exhibited relatively uniform printing quality (Figure 8c). These results demonstrate that AcTPB meets the requirements for 3D printing and holds potential for application in the 3D printing of ultra-low-temperature solid propellants.

## 4. Conclusions

In summary, high cis-1,4 content hydroxyl-terminated polybutadiene (cis-HTPB) with tailored molecular weights was successfully synthesized via oxidative cleavage. The obtained cis-HTPB maintains a high cis-1,4 content of 93% and an ultra-low glass transition temperature near −100 °C, conferring outstanding low-temperature flexibility. Furthermore, acrylate modification of cis-HTPB yielded a UV-curable derivative (AcTPB), which still retains the low Tg (around −100 °C) after curing and could be used for 3D printing technology.

## Figures and Tables

**Table 1:** The specific content of the mCPBA in the three prepared cis-HTPB.

Sample	mCPBA: Butadiene Units	mCPBA:H_5_IO_6_	mCPBA:NaBH_4_
Cis-HTPB-1	3%	1:1	1:4
Cis-HTPB-2	6%	1:1	1:4
Cis-HTPB-3	9%	1:1	1:4

**Table 2:** Molecular weight of BR and cis-HTPB.

Sample	M_n_	M_w_	M_v_	PDI
BR	173,674	609,278	499,703	3.51
Cis-HTPB-1	5831	17,502	14,905	3.00
Cis-HTPB-2	3215	9121	7816	2.84
Cis-HTPB-3	2187	7116	6060	3.25

## Data Availability

The original contributions presented in this study are included in the article. Further inquiries can be directed to the corresponding authors.
